# Copy Number Variation Affecting the *Photoperiod-B1* and *Vernalization-A1* Genes Is Associated with Altered Flowering Time in Wheat (*Triticum aestivum*)

**DOI:** 10.1371/journal.pone.0033234

**Published:** 2012-03-22

**Authors:** Aurora Díaz, Meluleki Zikhali, Adrian S. Turner, Peter Isaac, David A. Laurie

**Affiliations:** 1 John Innes Centre, Norwich Research Park, Norwich, Norfolk, United Kingdom; 2 iDNA Genetics Ltd., The Norwich BioIncubator, Norwich Research Park, Norwich, United Kingdom; University of Massachusetts Amherst, United States of America

## Abstract

The timing of flowering during the year is an important adaptive character affecting reproductive success in plants and is critical to crop yield. Flowering time has been extensively manipulated in crops such as wheat (*Triticum aestivum* L.) during domestication, and this enables them to grow productively in a wide range of environments. Several major genes controlling flowering time have been identified in wheat with mutant alleles having sequence changes such as insertions, deletions or point mutations. We investigated genetic variants in commercial varieties of wheat that regulate flowering by altering photoperiod response (*Ppd-B1* alleles) or vernalization requirement (*Vrn-A1* alleles) and for which no candidate mutation was found within the gene sequence. Genetic and genomic approaches showed that in both cases alleles conferring altered flowering time had an increased copy number of the gene and altered gene expression. Alleles with an increased copy number of *Ppd-B1* confer an early flowering day neutral phenotype and have arisen independently at least twice. Plants with an increased copy number of *Vrn-A1* have an increased requirement for vernalization so that longer periods of cold are required to potentiate flowering. The results suggest that copy number variation (CNV) plays a significant role in wheat adaptation.

## Introduction

Cultivated bread wheat (*Triticum aestivum*) is an allohexaploid combining A, B and D genomes from three diploid ancestors [1]. The wild diploids and wheat in its original domestic form are winter annual long day plants [2], germinating in autumn and requiring an extended period of cold (vernalization) before they can respond to the flowering stimulus of increasing day length (photoperiod) in the following spring. These requirements have been altered during domestication to adjust the life cycle to local conditions, enabling wheat to be highly productive in a wide range of environments.

Wheat's ancestors and many modern varieties are described as photoperiod sensitive. That is, they flower rapidly in long days but are late flowering in short days. Day neutral (photoperiod insensitive) varieties flower rapidly in short or long days. This allows production in environments where appropriate temperature and rainfall coincide with short day conditions or where early flowering avoids high summer temperatures, as in Southern Europe [3]. The day neutral phenotype was an important component of the “Green Revolution” and continues to be widely used globally. It results from semi-dominant mutations at one or more of the collinear (homoeoallelic) *Photoperiod-1* (*Ppd-1*) loci on chromosomes 2A, 2B and 2D. The *Ppd-1* genes are members of the pseudo-response regulator (*PRR*) family orthologous to the *Ppd-H1* gene of barley [4], [5].

Four day neutral alleles have been characterized in wheat previously. They are given an *a* suffix (*Ppd-A1a*, *Ppd-B1a* and *Ppd-D1a*) while wild type alleles have a *b* suffix [6]. Three *Ppd-A1a* alleles and the single *Ppd-D1a* allele are deletions that remove a shared region of the promoter likely to be important for regulation [5], [7].

*Ppd-1b* (wild type) alleles have a marked diurnal fluctuation in expression, with very low transcript levels at dawn, a peak 3–6 h after dawn and a subsequent drop to very low levels in the dark. *Ppd-1a* alleles that have been studied show elevated expression throughout the 24 h period but particularly at dawn and during the dark period. This is associated with expression of *TaFT1* (the wheat orthologue of the flowering inducer *FLOWERING LOCUS T*) in short days [5], [7]. This suggests that day neutral mutations share the property of mis-expression of *Ppd-1* and the induction of *TaFT1* without the normal long day cue.

We investigated *Ppd-B1a* alleles identified by genetic mapping in five diverse sources: ‘Chinese Spring’ (China [8], [9]), ‘Sonora64’ (Mexico [10]), ‘Timstein’ (Australia [9]), ‘C591’ (India [11]) and ‘Récital’ (France [12]). However, sequencing *Ppd-B1* from ‘Chinese Spring’, ‘Sonora64’ and ‘Récital’ revealed no candidate mutation [5]. This paper characterizes the mutations in previously unresolved *Ppd-B1a* alleles and their effects on gene expression.

Vernalization is another important flowering time control. The ancestors of wheat and many modern varieties are winter types that require vernalization, but during domestication there has been selection for spring types that do not. Spring types result from mutation of the *Vernalization-1* (*Vrn-1*), *Vrn-2* or *TaFT1* (also called *Vrn-3*) genes [13]–[16]. *Vrn-1* encodes a MADS-box transcription factor whose expression increases quantitatively during vernalization. This potentiates flowering by inhibiting expression of the flowering repressor *Vrn-2* [17], [18]. Semi-dominant mutations at one or more of the homoeoallelic *Vrn-A1*, *Vrn-B1* or *Vrn-D1* genes (chromosomes 5A, 5B and 5D, respectively) are the predominant cause of spring types in wheat [16].

In addition to the winter/spring difference, winter types vary in the duration of cold necessary to complete vernalization. We refer to this as variation in vernalization requirement. This aspect of vernalization is much less studied, but recently it was proposed that *Vrn-A1* was the cause; specifically a single base difference in exon 4 [19]. We investigated three UK winter wheat varieties with short, medium and long vernalization requirements, respectively. Genetic mapping, sequencing and analysis of gene expression also identified *Vrn-A1* as the cause but in contrast to [19] the exon 4 difference did not distinguish the three types.

For the *Ppd-B1a* and *Vrn-A1* alleles we investigated, the changes in flowering time were associated with increased gene copy number and not with specific sequence polymorphisms. Copy number variation (CNV) is a type of mutation that has been widely studied in human genetics [20], [21] and is well recognized in studies at the whole genome level in plants [22], [23] although those studies include changes arising from segemental duplications, differences between homoeologous chromosomes and variation within gene families. Recent whole genome sequence and gene expression analysis of *Arabidopsis thaliana* accessions [24], [25] where it was shown that 9% of genes showed CNV between at least two of the 19 accessions studied but many of these are probably pseudogenes as only 388 were expressed [25]. Of these, 237 (61%) were differentially expressed and for 54 (14%) this was attributed to CNV, leading to the conclusion that CNV has a modest effect on gene expression and by extension would have little impact on phenotype. In contrast, work on CBF genes in barley in relation to abiotic stress [26] and our studies on flowering time started with phenotypic variation and identified independent CNV alleles. This suggests that CNV may be the cause of important adaptive variation.

## Results

### Flowering time phenotypes of *Ppd-B1a* alleles

**Figure 1:**
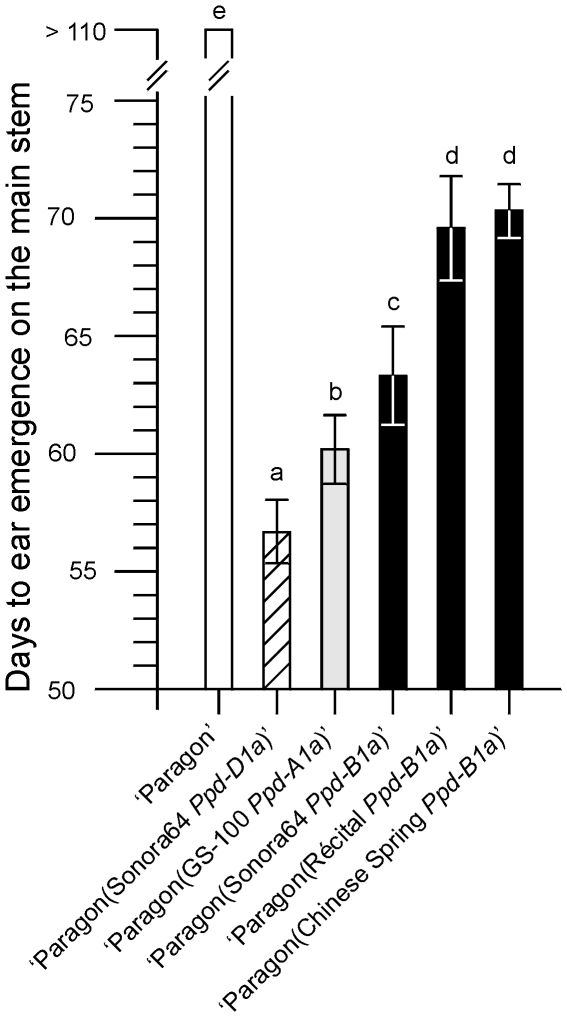
Flowering time phenotypes of *Ppd-1a* introgression lines. Means and standard deviations of days to ear emergence for plants grown in short days (10 h natural light). ‘Paragon’ control (white; n = 10). The mean of two independent families (n = 20 in total) is shown for *Ppd-D1a* (hatched), *Ppd-A1a* (grey) and three *Ppd-B1a* alleles (black). Means significantly different by t-test (p<0.05) have different letters.

Firstly, we describe our analysis of *Ppd-B1*. The photoperiod sensitive hexaploid spring wheat variety ‘Paragon’ was the recurrent parent for introgression lines containing *Ppd-1a* alleles from ‘Chinese Spring’, ‘Sonora64’ or ‘Récital’. Two independent lines of each allele were developed and from each of these ten plants at BC_3_F_4_ (‘Récital’ *Ppd-B1a* allele) or BC_4_F_4_ (other alleles) were grown in short days (10 h natural light) in a photoperiod glasshouse. Days to ear emergence on the main stem were recorded for each plant (Figure 1). Plants with the ‘Sonora64’ *Ppd-B1a* allele flowered earlier than those with the ‘Chinese Spring’ or ‘Récital’ *Ppd-B1a* alleles. Plants with *Ppd-A1a* or *Ppd-D1a* alleles flowered earlier than those with a *Ppd-B1a* allele, consistent with [27], showing that these *Ppd-B1a* alleles had comparatively weak phenotypes. This contrasts with the *Ppd-B1a* allele described in [28] which has a phenotype comparable to *Ppd-D1a*.

### Copy Number Variation (CNV) at the *Ppd-B1* locus

It was previously shown that ‘Chinese Spring’ has a truncated *Ppd-B1* gene in addition to an intact version [5]. These are in close proximity because both are present on individual BAC clones. To determine copy number accurately a *Ppd-B1* specific quantitative TaqMan® assay was developed using a region present in the intact and truncated copies. Part of the wheat *CONSTANS2* gene (*TaCO2*, also called *TaHd1* [29]) from the group 6 chromosomes was used as an internal control. Nullisomic-tetrasomic lines of ‘Chinese Spring’ verified the assay.

**Figure 2:**
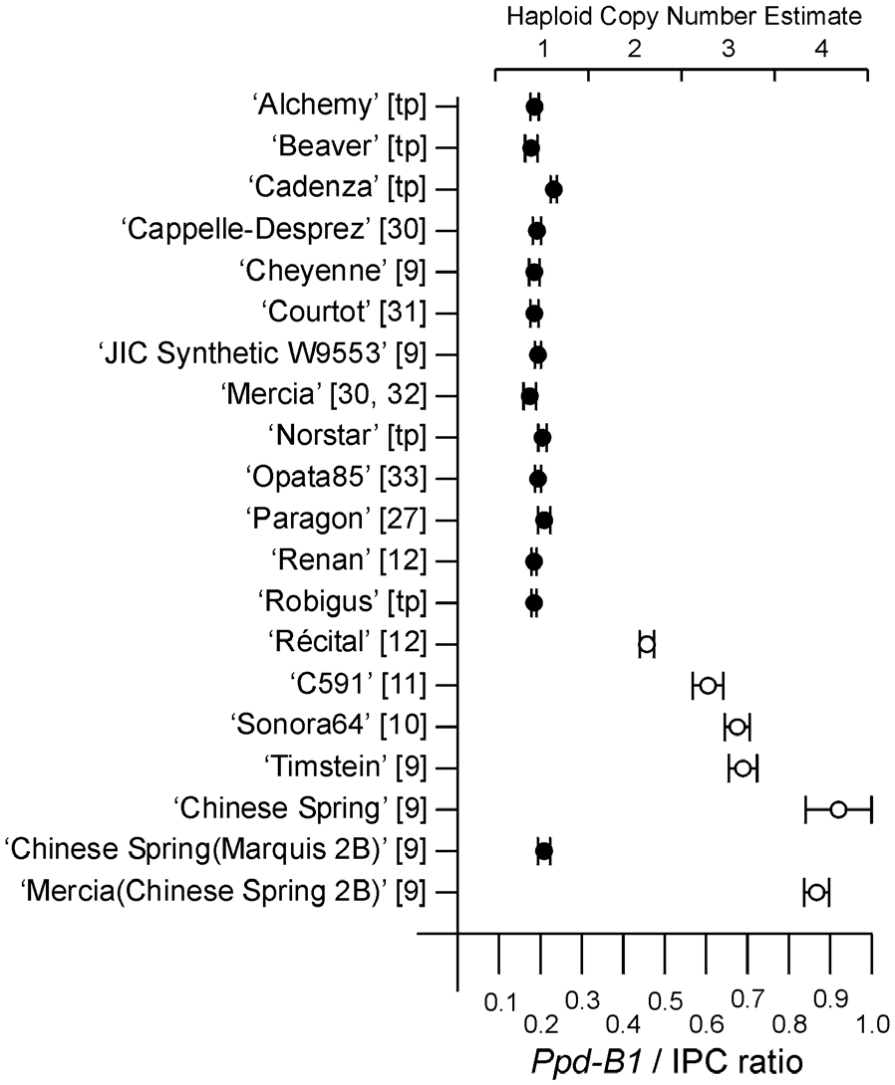
TaqMan® estimates of *Ppd-B1* haploid copy number. Solid circles are genotypes known to have a photoperiod sensitive (*Ppd-B1b*) allele and open circles are genotypes known to have a day neutral (*Ppd-B1a*) allele. References are [tp] this paper, [30]–[33] and others described in the text. Copy number was estimated from the *Ppd-B1*/Internal Positive Control (IPC) signal ratio. Means and standard deviations of four measurements are shown. T-tests showed the classes differed significantly from one another (p<0.001).

*Ppd-B1b* genotypes had a haploid copy number of one (i.e. one copy per 2B chromosome), while ‘Chinese Spring’ had four copies. Furthermore, the other genotypes with a day neutral *Ppd-B1a* allele also had elevated copy numbers; two in ‘Récital’ and three in ‘Sonora64’, ‘Timstein’ and ‘C591’ (Figure 2). A ‘Mercia(Chinese Spring 2B)’ single chromosome substitution line in which the ‘Chinese Spring’ 2B (carrying *Ppd-B1a*) replaced the ‘Mercia’ 2B had the same increased copy number as ‘Chinese Spring’ itself. Conversely, a line in which the 2B chromosome from the photoperiod sensitive variety ‘Marquis’ (*Ppd-1Bb*) replaced the ‘Chinese Spring’ chromosome had a low copy number, showing that all the additional gene copies were on chromosome 2B. These data suggested that increased copy number could be the basis of the *Ppd-B1a* alleles.

### The ‘Chinese Spring’ type *Ppd-B1a* allele

**Figure 3:**
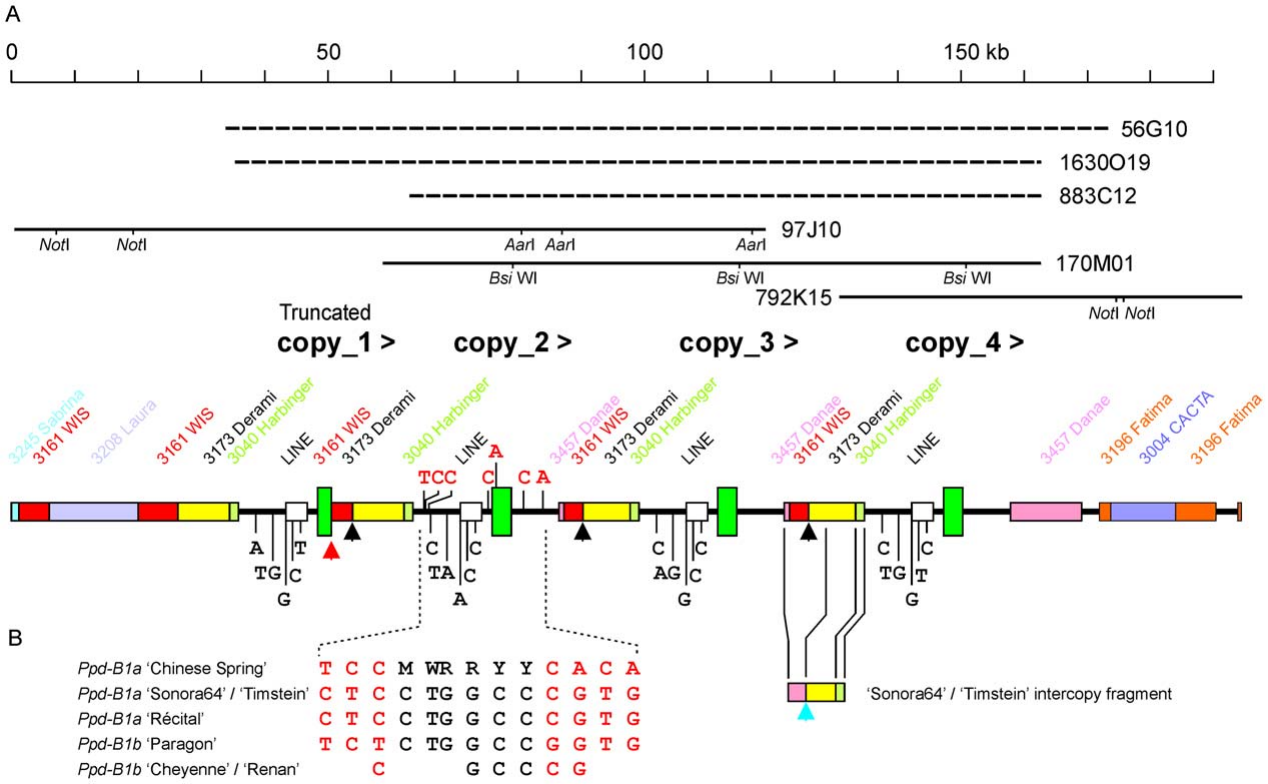
Structure of the ‘Chinese Spring’ *Ppd-B1a* allele and sequence haplotypes from other alleles. A) A 185 kb region containing ‘Chinese Spring’ *Ppd-B1a* allele. Sizes and positions of fully sequenced BACs are shown by solid lines with additional sized and end sequenced BACs as dotted lines. *Ppd-B1* copies (exons plus introns; > shows 5′ to 3′ orientation) are large dark green rectangles and the solid black line shows upstream and downstream regions with no homology to known repeated sequences. Small coloured rectangles show transposable elements annotated using the TREP database (http://wheat.pw.usda.gov/ITMI/Repeats/). Vertical arrows show the junction between intact copies (black) or between the gene and transposon in the truncated copy (red). B) Haplotypes of *Ppd-B1* alleles. Intervarietal SNPs are in red. These were invariant between copies in ‘Chinese Spring’ and for clarity only the positions in copy_2 are shown. Bases distinguishing the copies in ‘Chinese Spring’ are in black (IUPAC codes). Coloured rectangles in the ‘Sonora64’/‘Timstein’ intercopy fragment are transposable elements as in (a). Vertical blue arrow shows the junction between copies. Junction sequences are given in Figure S2.

A BAC library [34] allowed the ‘Chinese Spring’ allele to be fully described. Three overlapping B genome BAC clones (97J10, 170M01 and 792K15) were sequenced and three additional BACs (56G10, 883C12 and 1630O19) were analyzed by end sequencing, restriction digestion and pulse-field gel electrophoresis (PFGE) (Figure 3A). These BACs were originally selected by filter hybridization [5]. They were further classified by restriction mapping and PCR to identify BACs containing the truncated copy.

Sequence assembly from 792K15 was straightforward and showed a single intact copy of the *Ppd-B1* gene flanked by retrotransposons. 97J10 and 170M01 did not assemble readily because of sequence differences at the ends of approximately 35 kb regions containing either an intact or truncated gene. Digestion with *Not*I allowed BAC clone inserts to be sized. 170M01, which did not contain the truncated copy, would contain three repeat units of approximately 35 kb. PFGE of 97J10 using *Aar*I, which does not cut within the truncated copy, placed the truncated copy at the left end of the repeating structure (copy_1), showing that the *Ppd-B1a* locus of ‘Chinese Spring’ comprised one truncated copy and three intact copies of the *Ppd-B1* gene, all in the same orientation, within a 185 kb region (Figure 3A; JF946485).

An A/G SNP in exon 3 identified by [5] distinguished the *Ppd-B1a* allele of ‘Chinese Spring’ (A form) from the other varieties in Figure 2. Sequence trace files from the different BAC clones and a *Tsp*509I CAPS assay of amplicons from ‘Chinese Spring’ and individual BAC clones showed that all copies had the A form. Although exon and intron sequences were identical apart from the truncation in copy_1, different haplotypes were found upstream as shown by six nucleotides below each gene copy (Figure 3A). The haplotype found in 97J10 but not in 170M01 or 792K15 was assigned to the truncated copy and 792K15 gave the haplotype of copy_4. Restriction digestion and religation of 170M01 with *Bsi*WI recovered subclones containing the leftmost intact copy from which the haplotype of copy_2 was obtained. The remaining haplotype was assigned to copy_3. Haplotypes, BAC sizes and TaqMan® assays were all consistent with a copy number of four. A model for the origin of the ‘Chinese Spring’ allele is shown in Figure S1. We suggest that breakage and repair generated a chromosome with two copies. Unequal crossing-over then generated three and four copy versions and partial deletion of one copy at an intermediate or final stage gave the current structure.

The junction between intact gene copies was verified by PCR amplification and sequencing and used to design a diagnostic PCR assay (Figure S2). This produced an amplicon from ‘Chinese Spring’ and lines into which the ‘Chinese Spring’ *Ppd-B1a* allele was introgressed. No amplicon was produced in other *Ppd-B1a* or photoperiod sensitive genotypes. A PCR assay for the gene/transposon breakpoint in the ‘Chinese Spring’ truncated copy gave the same result (Figure S2). The copy number of four, the intercopy break point, the exon 3 SNP and the partial deletion of one copy were therefore specific to the ‘Chinese Spring’ allele.

### The ‘Sonora64’ type *Ppd-B1a* allele

‘Sonora64’ and ‘Timstein’ had a haploid copy number of three (Figure 2). Partial sequence from the ‘Timstein’ allele (DQ885765) was previously reported [5]. The ‘Chinese Spring’ sequence was used to design additional PCR amplicons to extent the sequence further upstream and downstream and to sequence the ‘Sonora64’ allele (JF946486). The sequences were identical, gave no indication of variation between copies and confirmed that there was no mutation such as a promoter insertion or deletion likely to cause the day neutral phenotype. However, SNPs distinguishing the ‘Sonora64’/‘Timstein’ allele from ‘Chinese Spring’ were found (Figure 3B).

Long range PCR using primers at the ends of the gene sequence amplified an intercopy fragment of approximately 9 kb from ‘Sonora64’ (Figure 3B; JF946487). An identical sequence was obtained from ‘Timstein’. The fragment was composed of transposable elements previously found flanking the *Ppd-B1* gene in ‘Chinese Spring’. However, the breakpoint was different and the intercopy fragment lacked the WIS element of the ‘Chinese Spring’ intercopy sequence. A PCR assay for the ‘Sonora64’ breakpoint produced an amplicon from ‘Sonora64’, ‘Timstein’ and ‘C591’, the other variety with a copy number of three (Figure 2). No PCR product was produced from ‘Chinese Spring’ or ‘Récital’ (Figure S2). The different intercopy breakpoints and haplotypes show that the ‘Sonora64’/‘Timstein’/‘C591’ and ‘Chinese Spring’ alleles must have derived from independent amplification events.

### The ‘Récital’ type *Ppd-B1a* allele

‘Récital’ had a haploid copy number of two (Figure 2) and the same haplotype as ‘Sonora64’ and ‘Timstein’ (Figure 3B; DQ885763). However, long range PCR using a range of primers failed to amplify an intercopy fragment. Analysis of 143 BC_3_F_2_ plants in which the ‘Récital’ allele was introgressed into the photoperiod sensitive ‘Paragon’ background showed a 3∶1 segregation for early flowering in short days (10 h light) which was correlated with copy number, showing the copies to be genetically linked. This suggests that the ‘Récital’ allele is derived from a third independent amplification event or from the ‘Sonora64’/‘Timstein’ type by further mutation.

### The ‘Paragon’, ‘Cheyenne’ and ‘Renan’ *Ppd-B1b* alleles

PCR amplicons based on the ‘Chinese Spring’ sequence were also used to obtain extended sequence from ‘Paragon’ (DQ885762), ‘Cheyenne’ and ‘Renan’ which have wild type *Ppd-B1b* alleles (Figure 2, S2). This showed that no SNPs were consistently associated with the *Ppd-B1a* alleles (Figure 3B). This suggests that increased copy number may be the cause of the day neutral phenotype.

### *Ppd-B1* expression

**Figure 4:**
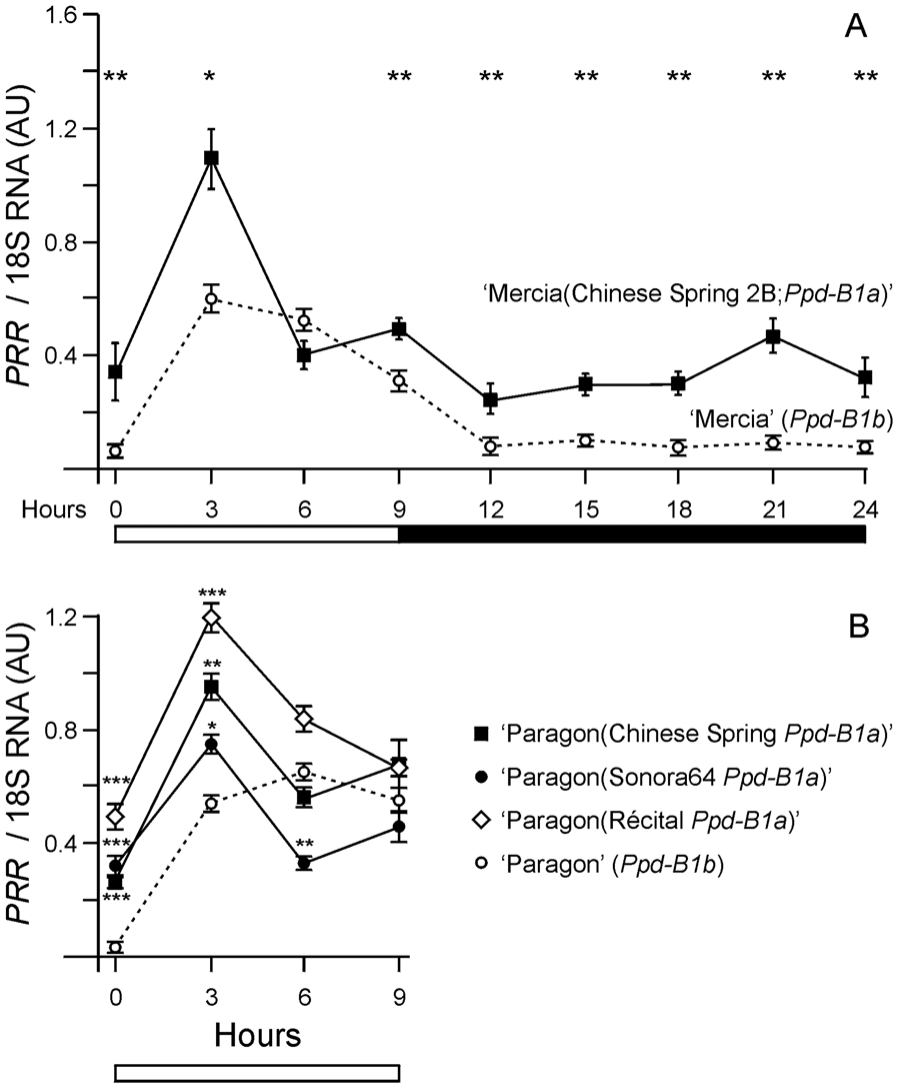
Quantitative RT-PCR analysis of *Ppd-B1* expression in short days. A) Gene expression in short days (light period 0–9 h; white bar) sampled at 3 h intervals over a 24 h time course comparing ‘Mercia’ (*Ppd-B1b* allele; dotted line, open circles) with a ‘Mercia(Chinese Spring 2B)’ single chromosome substitution line (*Ppd-B1a*; solid line, closed squares). B) Gene expression in short days (light period 0–9 h; white bar) sampled at 3 h intervals from 0 to 9 h comparing ‘Paragon’ (*Ppd-B1b*; dotted line) with BC_4_F_3_ introgression lines homozygous for *Ppd-B1a* alleles from ‘Chinese Spring’ or ‘Sonora64’ or BC_3_F_3_ introgression lines homozygous for *Ppd-B1a* from ‘Récital’ (solid lines). Each time point is the mean of three biological replicates normalized against expression of an 18S rRNA control. Expression is shown as arbitrary units (AU). Standard errors of means are indicated by error bars and asterisks show probabilities from one-way ANOVAs of expression levels comparing the respective *Ppd-B1a* alleles to the ‘Paragon’ *Ppd-B1b* allele (* p<0.5; ** p<0.01; *** p<0.001).

Expression was analyzed using a *Ppd-B1* specific quantitative RT-PCR assay. First, ‘Mercia’ (*Ppd-B1b*) was compared with a ‘Mercia(Chinese Spring 2B)’ (*Ppd-B1a*) single chromosome substitution line. Plants were grown in short days (9 h light; 16°C) for 21 days and RNA samples were taken from three biological replicates, each of four plants, at 3 h intervals over a 24 h period. Expression of the ‘Mercia’ allele was similar to that of *Ppd-1b* alleles in previous studies [5], [7] with very low expression at dawn and a peak of expression 3–6 h after dawn. The ‘Chinese Spring’ *Ppd-B1a* allele had significantly higher levels at dawn, a higher expression peak, and elevated expression throughout the dark period (Figure 4A). This change was similar to that found for *Ppd-A1a* and *Ppd-D1a* promoter deletion alleles [5], [7] but was less extreme, consistent with the weaker phenotype of the ‘Chinese Spring’ *Ppd-B1a* allele (Figure 1).

There was no sequence polymorphism between the expressed regions of the intact gene copies so it was not possible to determine if all were expressed. However, RT-PCR using a forward primer in exon 6 and a reverse primer in the WIS retrotransposon produced a product with intron 6 correctly spliced, showing that the truncated copy was expressed. The transcript had a stop codon 2 bp beyond the junction which would generate a protein lacking the conserved CCT domain and which is likely to be non-functional.

Subsequent development of ‘Paragon’ introgression lines with different *Ppd-B1a* alleles allowed an additional comparison. Plants of ‘Paragon’ and ‘Paragon(Chinese Spring *Ppd-B1a*)’, ‘Paragon(Sonora64 *Ppd-B1a*)’ and ‘Paragon(Récital *Ppd-B1a*)’ introgression lines were grown in short days and sampled at the end of the dark period and at three time points in the lit period as described above (Figure 4B). This showed that increased copy number of *Ppd-B1* was always associated with significantly elevated gene expression, particularly at dawn when expression in the wild type was very low.

Having identified copy number variation at the *Ppd-B1* locus and described its relationship to photoperiod response, we now consider variation in vernalization requirement, which is another important flowering time control.

### Flowering time after different vernalization treatments

The winter wheat varieties ‘Claire’, ‘Malacca’ and ‘Hereward’ require short, medium or long low temperature treatments, respectively, to achieve full vernalization. To study this quantitatively, plants were vernalized at 7°C in 8 h days in increments of 1 week for 0 to 10 weeks (‘Hereward’) or 0 to 6 weeks (‘Claire’ and ‘Malacca’) after which they were transferred to a heated lit glasshouse providing long days (18 h light). Day length was sufficient to satisfy photoperiod response, and genotyping showed that no *Ppd-1a* allele was present.

**Figure 5:**
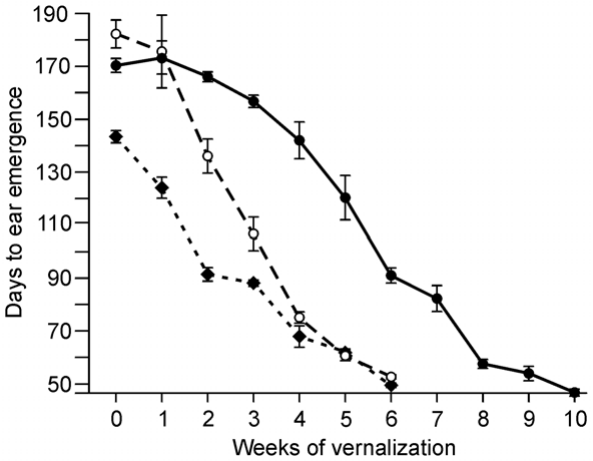
Days to ear emergence for winter wheat plants after different vernalization times. Plants were vernalized (7°C; 8 h light) for 0 to 6 weeks (‘Claire’ – solid diamonds, dotted line; ‘Malacca’ – open circles, dashed line) or 0 to 10 weeks (‘Hereward’ – solid circles, solid line) and days to ear emergence on the main stem were recorded. Each point is the mean of five plants. Bars show standard deviations.

Days to ear emergence on the main stem were recorded for five plants of each genotype per time point. Unvernalized ‘Claire’ plants flowered about 40 days before ‘Malacca’ but the acceleration of flowering per week of vernalization was greater in ‘Malacca’ so that flowering times were similar after 4 or more weeks of cold. Unvernalized plants of ‘Hereward’ and ‘Malacca’ flowered at a similar time but the 1 to 4 week treatments in ‘Hereward’ had less effect on flowering, after which the response was similar to ‘Malacca’ (Figure 5).

### Genetic mapping of vernalization requirement

To investigate the genetic basis of long vernalization requirement in ‘Hereward’ we selected a 4 week vernalization treatment (7°C, 8 h light) to study a population of 77 doubled haploid (DH) lines from a ‘Malacca’×‘Hereward’ cross. Three plants of each DH line and four plants of each parent were vernalized and then grown in a heated glasshouse with supplementary lighting providing long days (18 h light). Days to ear emergence on the main stem were recorded and averaged for each genotype. QTL mapping showed one significant peak (LOD>22.0; Figure S3) associated with barc151, an SSR marker linked to *Vrn-A1* on chromosome 5A [35].

### Copy Number Variation at the *Vrn-A1* locus

Approximately 12 kb of *Vrn-A1* sequence including all exons and introns was obtained from ‘Malacca’ (JF965396) and ‘Hereward’ (JF965397) by direct sequencing of overlapping PCR amplicons generated from gene specific primers based on the ‘Triple Dirk C’ winter allele (AY747601 [16]). No differences previously associated with spring alleles were found and no sequence difference was found between ‘Malacca’ and ‘Hereward’. However, both consistently gave an anomalous C/T double peak in sequence trace files from the amplicon covering exon 4 (Figure S4). Cloning and sequencing of the amplicon gave individual clones with either a C or a T, showing that two versions of exon 4 were present. Chi^2^ analyses showed that the number of C and T clones for ‘Malacca’ (63∶73) only fitted a 1∶1 ratio while for ‘Hereward’ (51∶87) they only fitted a 1∶2 ratio, suggesting two and three copies of *Vrn-A1*, respectively. Full length cDNA clones from RNA of vernalized ‘Malacca’ and ‘Hereward’ plants also had the C and T forms showing that two intact variants of the gene were present and expressed. The C/T difference in exon 4 is likely to be that previously reported as distinguishing ‘Jagger’ from ‘2174’ [19]. Our results differ in that both sequences were found within ‘Malacca’ and ‘Hereward’. The presence of C and T sequences was not due to heterozygosity or mixed seed stocks as it was consistent and did not segregate in progeny plants.

**Figure 6:**
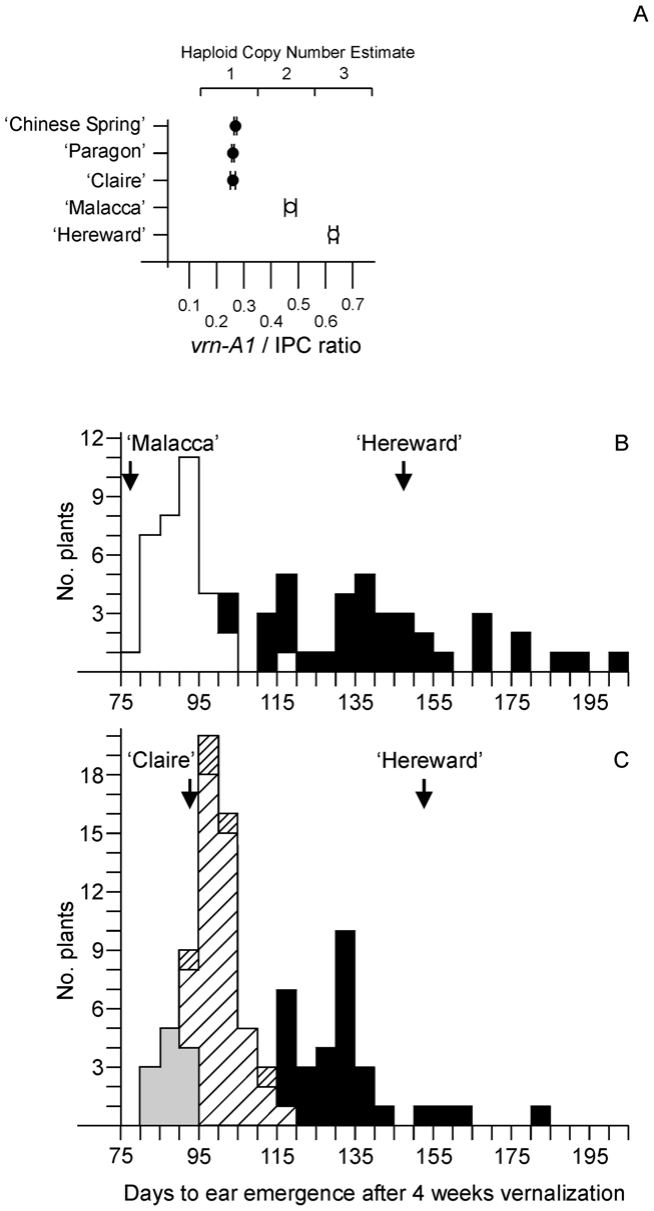
TaqMan® estimates of *Vrn-A1* haploid copy number and days to ear emergence in winter wheat mapping populations. A) Copy number estimated from the *Vrn-A1*/Internal Positive Control (IPC) signal ratio. Solid circles are genotypes estimated to have one copy of *Vrn-A1*. Open circles are genotypes estimated to have two or three copies. Means and standard deviations of four measurements are shown. . T-tests showed the classes differed significantly from one another (p<0.01). B) Days to ear emergence in ‘Malacca’×‘Hereward’ DH lines vernalized for 4 weeks (7°C; 8 h light) and grown in a heated, lit glasshouse providing 18 h light. The score for each DH line was the mean of three plants. Lines with two copies of *Vrn-A1* in white, those with three are in black. Arrows show flowering times of the parental varieties (mean of four plants). C) Days to ear emergence in ‘Claire’×‘Hereward’ F_2_ plants vernalized for 4 weeks (7°C; 8 h light) and grown as in (b). Plants homozygous C (exon 7 SNP) and with one copy of *Vrn-A1* are in grey. Plants heterozygous C/T and with intermediate *Vrn-A1* copy number are hatched (double hatched squares shows five plants with ambiguous copy number estimates between intermediate and high). Plants homozygous T and with three copies of *Vrn-A1* are in black. Arrows show flowering times of the parental varieties (mean of four plants).

A TaqMan® assay specific for *Vrn-A1* (using the same internal control employed for *Ppd-B1*) confirmed a haploid copy number of two for ‘Malacca’ and three for ‘Hereward’ (Figure 6A). The ‘Malacca’×‘Hereward’ DH population showed a 1∶1 ratio for copy number showing that the copies were linked in both varieties and behaved as single genetic entities. When *Vrn-A1* was scored qualitatively by copy number the QTL peak coincided with *Vrn-A1* (Figure S3) and higher copy number was associated with late flowering after 4 weeks vernalization (Figure 6B). These results show that ‘Malacca’ has a duplication of the *Vrn-A1* gene with one copy having a C in exon 4 and one copy a T. ‘Hereward’ has one copy of the C form and two copies of the T form which could be the result of unequal crossing over or an additional breakage and rejoining event. Long range PCR was attempted between copies but no intercopy fragment was obtained.

‘Malacca’ and ‘Hereward’ had the same haplotype as ‘IL369’ (AY747599). The latter has a spring allele of *Vrn-A1* [36] with a deletion in intron 1 but also an intact intron 1 sequence, suggesting a duplication [16]. We found that ‘IL369’ had a copy number of two, suggesting it derived from the ‘Malacca’ type winter allele, and copy specific PCR amplicons showed that the intact and deletion copies had a C and T in exon 4, respectively (Figure S5B). As an intron 1 deletion is considered sufficient to induce expression and confer a spring growth habit it is likely that the T form is functional even though it changes a conserved amino-acid in the K domain of the VRN-A1 protein (Figure S5B [37]).

‘Claire’ had a haploid copy number of one (Figure 6A) and its sequence (JF965395) had a different haplotype to ‘Malacca’ and ‘Hereward’. Exon 4 in ‘Claire’ was the C type and there was an additional exon 7 SNP which was C in ‘Claire’ and T in ‘Malacca’ and ‘Hereward’ (i.e. a T was present in all the gene copies). This enabled a ‘Claire’×‘Hereward’ F_2_ population to be used for an additional test of the genetic behaviour of the ‘Hereward’ *Vrn-A1* copies (Figure 6C).

Ninety-six F_2_ plants and four plants of each parent were given 4 weeks vernalization and grown as described above. Days to ear emergence on the main stem were recorded. Although there was a deficiency of ‘Claire’ type homozygotes there was a clear association of the exon 7 SNP with flowering time. Early flowering plants were homozygous for the ‘Claire’ allele while heterozygotes had an intermediate flowering time and ‘Hereward’ homozygotes flowered later. Very similar results were obtained when the population was analyzed using the TaqMan® assay (Figure 6C). This is consistent with linkage of the ‘Hereward’ copies and, as with the ‘Malacca’×‘Hereward’ data, increased copy number of *Vrn-A1* was strongly correlated with later flowering.

### *Vrn-A1* expression

*Vrn-1* expression increases quantitatively during vernalization [13], [17], [18]. To compare ‘Claire’, ‘Malacca’ and ‘Hereward’, seedlings were harvested at the end of each week in the same experiment used to measure flowering time. *Vrn-A1* expression was measured by gene specific quantitative RT-PCR.

**Figure 7:**
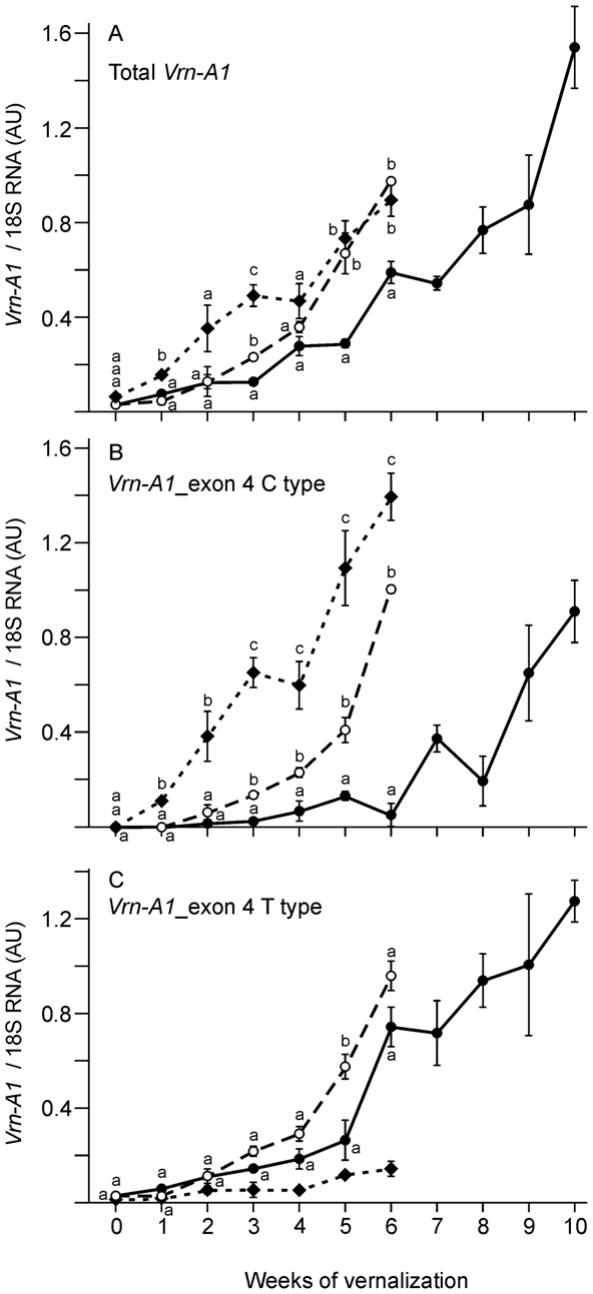
Quantitative RT-PCR analysis of *Vrn-A1* expression level in winter wheat plants vernalized for different times. Plants were vernalized (7°C; 8 h light) for 0 to 6 weeks (‘Claire’ – solid diamonds, dotted line; ‘Malacca’ – open circles, dashed line) or 0 to 10 weeks (‘Hereward’ – solid circles, solid line). A) Total *Vrn-A1* expression (C and T forms) in leaf samples at the end of the vernalization period or in unvernalized 7 day old seedlings for the 0 weeks treatment. Each time point is the mean of three biological replicates normalized against expression of an 18S rRNA control. Expression is shown as arbitrary units (AU). Bars show standard errors of means. B) Expression of the *Vrn-A1* form with the C in exon 4. C) Expression of the *Vrn-A1* form with the T in exon 4. The low level signal in ‘Claire’, which lacks the T form, was attributed to mis-priming of the assay in the absence of the correct template. At each time point letter b and c indicate significant differences (p<0.05 by ANOVA) from the ‘Hereward’ level (a).

Total *Vrn-A1* expression (combining the exon 4 C and T forms) increased most rapidly in ‘Claire’ and was slowest in ‘Hereward’, while ‘Malacca’ was intermediate (Figure 7A). The C and T forms were also analyzed separately. The C form, which encodes the typical form of the protein, gave the clearest difference between the varieties and was most rapidly induced in ‘Claire’ and most slowly in ‘Hereward’, with ‘Malacca’ intermediate (Figure 7B). Similarly, the T form was more rapidly induced in ‘Malacca’ than in ‘Hereward’ (Figure 7C). Irrespective of whether the T form affects flowering, all the assays showed a trend that increased copy number was associated with slower induction of expression, consistent with the observed differences in flowering time.

## Discussion

Changes in flowering time in wheat have previously been shown to be caused by sequence variation such as insertions, deletions and point mutations in the *Ppd-1* and *Vrn-1* genes. We show that flowering time variation is also associated with these genes when they show CNV but no sequence changes likely to affect function. CNV was consistently associated with altered gene expression and it is highly unlikely that CNV in independent alleles of *Ppd-B1* and in *Vrn-A1* would occur by chance. Taken together the observations suggest that CNV was a plausible cause of the observed flowering time variation.

CNV is a significant source of polymorphism in the human genome [20], [21] but is less studied in plants, particularly in crops. However, a barley variety with increased boron toxicity tolerance had an increased copy number of a boron transporter [38] and increased frost tolerance in barley is associated with increased *CBF* gene copy number at the *Fr-2* locus [26]. Variation has also been reported for the *Vrn-2* locus in tetraploid wheat but it is not clear if this affects phenotype [39]. In the case of *CBF* the variation is found within a large cluster of around ten related genes [36], while *Vrn-2* is normally found in tandem with one or two closely related genes [14], [39]. In these cases the presence of related sequences may favour CNV by unequal crossing over. In contrast, CNV for *Ppd-B1* and *Vrn-A1* affects genes that are single copy in wild type plants. It is likely that CNV alleles first arose by double-strand breakage and repair. Beneficial phenotypes resulting from altered flowering time would have been selected by farmers and phenotypes could have been enhanced by selecting higher copy numbers that resulted from unequal crossing-over.

For both genes we examined increased copy number was always associated with altered gene expression. *Vrn-A1* expression is very low in germinating seedlings and increases quantitatively during vernalization [13]. *Vrn-A1* was studied over 6 or 10 weeks and this showed that increasing copy number was found in alleles that required progressively more exposure to cold to become active (Figures 5, 6, 7). This was strongly correlated to differences in flowering time.

For *Ppd-B1* such a clear correlation was not observed (Figure 4B) but all alleles with increased copy number showed a deregulated pattern of expression and significantly increased expression at times when the transcript level of wild type alleles was very low. This was similar to changes in *Ppd-A1a* and *Ppd-D1a* alleles that confer a day neutral phenotype as a result of promoter mutations [5], [7] although the level of change in *Ppd-B1a* alleles was less, consistent with their weaker phenotypes (Figure 1). A tandem organization of CNV alleles predicts that they will not be completely stable but will give recombinants with higher or lower copy number. These will be very valuable for further study of the interaction between copy number and expression and for exploring the mechanism by which CNV might act. Possibilities include the titration of regulating proteins and, or, changes in chromatin organization. To resolve the mechanisms it is desirable to identify or create alleles in which the different gene copies can be distinguished.

Results from *Ppd-B1* prompted us to re-examine *Ppd-A1a* and *Ppd-D1a* alleles using TaqMan® assays, but we found no evidence for CNV. We therefore conclude that promoter mutation and CNV are two distinct mutational mechanisms leading to day neutrality. It is not clear why CNV only affects the *Ppd-B1* locus. This may be chance and CNV for the A and D genomes might be found in more extensive screens. Similarly, CNV may exist for *Vrn-B1* or *Vrn-D1* and it would therefore be of great interest to examine a wider range of germplasm.

We conclude that CNV is a significant source of variation for flowering time in wheat and may be an important but currently under recognized component of variation in general in crops. It will be important to consider CNV in germplasm screens and genetic mapping studies and to develop efficient markers for this type of polymorphism.

## Materials and Methods

### Plant materials

‘Mercia’ and ‘Mercia(Chinese Spring 2B)’ lines were from the John Innes Centre Germplasm Resources Unit. ‘Paragon(Récital *Ppd-B1a*)’ lines were developed as follows. ‘Récital’ carries *Ppd-B1a* and *Ppd-D1a* alleles [12]. Sixty-four ‘Paragon’×‘Récital’ BC_1_F_2_ plants were assayed for *Ppd-D1a* as described in [5] and *Ppd-D1a* carriers discarded. The remainder were grown in short days (10 h natural light). Early flowering plants would have *Ppd-B1a* and these were backcrossed to ‘Paragon’. BC_3_F_2_ families in short days showed 3∶1 segregation for flowering time and early flowering plants had higher *Ppd-B1* copy number, showing that *Ppd-B1a* was the major determinant of flowering time. BC_3_F_3_ families that were all early flowering in short days (10 h natural light) were selected. BC_3_F_4_ plants were compared to ‘Paragon’ and previously described ‘Paragon’ BC_4_F_4_ introgression lines homozygous for the ‘GS-100’ *Ppd-A1a*, ‘Chinese Spring’ *Ppd-B1a*, ‘Sonora64’ *Ppd-B1a* or ‘Sonora64’ *Ppd-D1a* alleles [27]. Plants were grown under summer conditions using a glasshouse with moving benches that travelled into a closed (dark) room after 10 h in the light.

### Standard PCR protocol

50 ng genomic DNA in 20 µl reactions comprising 1× PCR Buffer and 0.4 units *Taq* polymerase with 2 mM MgCl_2_, 250 nM of each primer and 200 µM dNTPs had 40 cycles of denaturing (95°C for 20 sec), annealing (55°C for 20 sec) and polymerisation (72°C for 1 min per kb of amplicon).

### Selection and sequencing of *Ppd-B1* BAC clones

Filter hybridization was used to select clones containing *Ppd-1* genes from a ‘Chinese Spring’ BAC library [5], [34]. B genome BAC clones selected by PCR using gene specific primers were sequenced by the commercial service of the Washington University in St. Louis Genome Sequencing Centre, 4444 Forest Park Boulevard, St. Louis MO 63108, USA. BAC sequence provided restriction endonuclease site positions. Restriction using *Bsu*36I or *Bsi*W1 followed by re-ligation and selection with chloramphenicol allowed recovery of the rightmost and leftmost *Ppd-B1* copies, respectively, in resulting subclones. The haplotypes of individual copies was determined by standard PCR (above) and Sanger sequencing (see sequencing of *Vrn-A1*).

### *Ppd-B1* intercopy sequencing

Long range PCR using primers facing outwards from the ends of the gene sequence (catttctcccgcttgtaagg and caacgttacagcttcggtca, respectively) was conducted using the Roche Expand Long template kit (Roche GMBH, 66298 Mannheim, Germany). This produced an approximately 9 kb fragment in ‘Sonora64’ and ‘Timstein’, which was analyzed by Sanger sequencing (below) using internal primers in a stepwise manner.

### PCR assays for junction sequences in *Ppd-B1a* alleles

PCR with “Hot Start” polymerase and buffer (Qiagen) and an initial denaturation step of 95°C for 5 minutes was used to detect; (a) the truncated *Ppd-B1* gene in the ‘Chinese Spring’ allele. Primers 219H05F2 (taactgctcctcacaagtgc) and 97J10R2 (ccggaacctgaggatcatc) gave a 425 bp product; (b) the junction between intact *Ppd-B1* copies in the ‘Chinese Spring’ allele. Primers PpdB1_F25 (aaaacattatgcatatagcttgtgtc) and PpdB1_R70 (cagacatggactcggaacac) gave a 994 bp product; (c) the junction between intact *Ppd-B1* copies in the ‘Sonora64’/‘Timstein’ allele. Primers PpdB1_F31 (ccaggcgagtgatttacaca) and PpdB1_R36 (gggcacgttaacacaccttt) gave a 223 bp product. Junction sequences and primer positions for assays are given in Figure S2.

### Sequencing of *Vrn-A1*

‘Triple Dirk D’ *vrn-A1* sequence (AY747601) was used to design a series overlapping PCR amplicons spanning the gene. Amplicons were obtained from genomic DNA using the standard PCR protocol and were directly sequenced using ABI Big Dye Mix v3.1 (Applied Biosystems Inc) under the manufacturer's conditions, with products resolved on an ABI 3730 capillary electrophoresis instrument. Segments of *Vrn-A1* from ‘IL369’ were amplified using primers IL369_F1 (tgaattcatgatcggagcag) and IL369_R1 (tgctgaacttctctgcaagtg) or IL396_F2 (tgctgaacttctctgcaagtg) and IL369_R1 (Figure S5) and directly sequenced.

### TaqMan® assays of copy number for *Ppd-1* and *Vrn-1*

20 µl reactions comprised water (3 µl), AbGeneQPCR mix (10 µl), Probe plus primers (2 µl) and DNA (5 µl). Samples were denatured at 95°C for 15 min followed by 40 cycles of [95°C for 15 sec, 60°C for 60 sec]. Forward and reverse primers for *Ppd-B1* were gcgtaagttactatctctcatggtgtatc and tttgttttagtacccagtaccataccag (0.2 µM) and the probe sequence was FAM-ctgctgcttcagttcctagtttcacttgtgtcc-TAMRA (0.1 µM). Forward and reverse primers for *Vrn-A1* were gcagcccacttttggtctcta and tctgccctctcgcctgtt (0.2 µM) and the probe sequence was FAM-tgtgttcgctttggttgtgcagca-TAMRA (0.1 µM). Forward and reverse primers for the *TaCO2* control were tgctaaccgtgtggcatcac and ggtacatagtgctgctgcatctg (0.1 µM) and the probe sequence was VIC-catgagcgtgtgcgtgtctgcg-TAMRA (0.1 µM). *Ppd-B1* plus control or *Vrn-A1* plus control were analyzed together in multiplexed reactions.

### Scoring Single Nucleotide Polymorphisms (SNPs) in *Vrn-A1*

Polymorphisms were scored using KBioscience KASP reagents (www.kbioscience.co.uk) in reactions containing water (2 µl), KASPar mix (4 µl), primers (0.1 µl), 50 mM MgCl_2_ (0.064 µl) and DNA (2 µl). An activation time (94°C, 15 min) was followed by 20 cycles of [94°C for 10 sec; 57°C for 5 sec; 72°C for 10 sec] followed by 24 cycles of [94°C for 10 sec; 57°C for 20 sec; 72°C for 40 sec]. Fluorescence was read as an end point reading at 25°C. Primer combinations were;

Exon4_C/T SNP specific primers: gaaggtgaccaagttcatgctaggcatctcatgggagaggatC, gaaggtcggagtcaacggattcaggcatctcatgggagaggatT (0.16 µM). Generic primer ccagttgctgcaactccttgagatt (0.4 µM).

Exon7_C/T SNP specific primers: gaaggtgaccaagttcatgctgagtttgatcttgctgcgccG, gaaggtcggagtcaacggattctgagtttgatcttgctgcgccA (0.16 µM). Generic primer cttccccacagctcgtggagaa (0.4 µM).

### Time course expression of *Ppd-B1*

RNA of ‘Mercia’ and ‘Mercia (Chinese Spring 2B)’ was extracted from seedlings grown for 20 days after germination in a controlled environment room with short days (9 h light; 16°C). Three replicate samples from each genotype were harvested into liquid nitrogen at each three-hourly time point over 24 h. RNA was sampled from 20 day old seedlings of ‘Paragon’ and ‘Paragon(Chinese Spring *Ppd-B1a*)’, ‘Paragon(Sonora64 *Ppd-B1a*)’ and ‘Paragon(Récital *Ppd-B1a*)’ introgression lines in the same way.

RNA was extracted using Trizol reagent (Invitrogen; http://www.invitrogen.com). DNA was removed by DNAseI digestion. cDNA was synthesized with Superscript II (Invitrogen) using the manufacturer's protocols with 5 µg of total RNA as template and a mixture of OligodT (12–18) (250 ng) and random hexamers (150 ng) as primers. 1/40 by volume of the final cDNA aliquot was used for real-time PCR as described below. In the case of 18S rRNA analysis, cDNA samples were diluted 1∶100 and 1/40 of this dilution was used as template.

Primers for *Ppd-B1* expression were agacgattcattccgctcc and agcagcaccatttgacagg (N.B these will detect transcripts from the intact and truncated copies in ‘Chinese Spring’). The forward primer was specific for *Ppd-B1*, and genome-specificity was confirmed using ‘Chinese Spring’ nullisomic-tetrasomic lines. The primers generate a 549 bp amplicon from cDNA. Similarly, primers taactgctcctcacaagtgc and ccggaacctgaggatcatc were used to detect expression of the truncated copy in ‘Chinese Spring’ (Figure S2). Quantitative RT-PCR was carried out essentially as in [7] (File S1).

### Quantification of *Vrn-A1* expression

Samples of leaf tissue from plants of each genotype/vernalisation time combination (three biological replicates per sample) were harvested in the vernalisation chamber at the end of each treatment 3–4 h after dawn and frozen in liquid nitrogen. RNA and cDNA were prepared as above, and *Vrn-A1* cDNA abundance was estimated using Real-Time RT-PCR (Supporting information). Primer pairs were:

*VrnA1*-all: cttgaacggtatgagcgctat and gcatgaaggaagaagatgaag, product size 396 bp.

*VrnA1*-exon 4 C form: gcatctcatgggagaggatC and gaatagtacgcctgtatgggctggat, product size 436 bp.

*VrnA1*-exon 4 T form: gcatctcatgggagaggatT and gaatagtacgcctgtatgggctggat, product size 436 bp. This assay produced a low level signal in ‘Claire’, which does not contain the T form (Figure 7C). We attribute this to mis-priming in the absence of a perfectly matching template.

## Supporting Information

Figure S1 **A model for the origin of the ‘Chinese Spring’** ***Ppd-B1a*** **allele and hypothetical structures of the ‘Sonore64’/‘Timstein’ and ‘Récital’** ***Ppd-B1a*** **alleles.** (A) The ‘Chinese Spring’ *Ppd-B1a* allele. (B) Hypothetical structure of the ‘Sonora64’/‘Timstein’ *Ppd-B1a* allele. (C) Hypothetical structure of the ‘Récital’ *Ppd-B1a* allele. *Ppd-B1* copies are shown as large dark green rectangles (exons plus introns), the solid black line shows upstream and downstream regions and small coloured rectangles show transposable elements as in Figure 3 of the main text.
(TIF)

Figure S2 **PCR assays detecting junction sequences in** ***Ppd-B1a*** **(day neutral) alleles and sequences of the junction regions.** (A) to (C) Reverse colour images of PCR products from genotypes with known *Ppd-B1* alleles. Genotypes with a known *Ppd-B1a* allele are boxed. ‘Chinese Spring(N2BT2D)’ is nullisomic for chromosome 2B (no *Ppd-B1* gene is present) and tetrasomic for chromosome 2D. ‘Chinese Spring(N2AT2B)’ is nullisomic for chromosome 2A and tetrasomic for chromosome 2B. (A) Assay for the gene/transposon junction (vertical arrow) in the truncated *Ppd-B1* copy of ‘Chinese Spring’. Primer positions for this assay are underlined in the sequence below. *Ppd-B1* gene sequence is to the left of the arrow (exons in uppercase, intron in lowercase) and the TREP 3161 WIS element is to the right of the arrow. A 425 bp band is produced when the junction is present. … TAACTGCTCCTCACAAGTGCCGGAAGGGAAAGACGCCGACCGTGAGAACGCCATGCCATATCTTGAGCTGAGCCTAAAGAGGTCGAGATCGACCACGGAGGGTGCGGATGCGATCCAGGAGGAACAGAGGAACGTCGTGAGACGATCAGACCTCTCGGCATTCACGAGgtgcaaagcataatatcagtgtcctttgtgaatccttaaatcatccatatgttgcatactaaccgttttcattctttgcaagGTACAATACGTGCTCGTTCTCCAATCAAGGCGGGGCAGGGTTCGTCGGGAGCTGTTCGCCCA↑cgtgactgccaagcgttcataacgtcttggttctatgggatgggtgcttcacctagcggtccttctaggacatatgctttcttggcagctatgaggatgatcctcaggttccgg . The same primers were used for RT-PCR where a 343 bp product was produced from cDNA. The product was cloned and sequenced, confirming that intron 6 was correctly spliced. The transcript has a stop codon (double underline) close to the break point which gives a predicted protein lacking a CCT domain. (B) Assay for the junction between intact *Ppd-B1* gene copies in the ‘Chinese Spring’ allele. Primer positions for this assay are underlined in the sequence below. The left primer spans the junction between *Ppd-B1* gene sequence and the TREP 3457 Danae element. The junction (vertical arrow) marks the start of the TREP 3161 WIS element sequence which contains the right primer. A 994 bp band is generated when the junction is present. …cacacaagcattatacctggaaccgtagcataattttcctcaacattttgagctacgtgtatggagtttgcattgttatcttcaacatttctcccgcttgtaaggcctgcaaaacattatgcatatagcttgtgtcggtgtacaaaagtaggagctctgcttttgacccctttacttgtgcacgagcagtcagagccacccgccacggccacgcataacagggcagggaagggaagccggagagaagccgaaggcacaagacaaacaaagcaacgacaaggaccaaggctacaaagtgcagatgggcgaagcaggtttcccctgcaagacccttgccgggggcagcctccgcagccccggcaagcccctttgccggggcaactcgcccacaccaatggagagagccacccttgaacccacggcctccaatgtcaaccaccacgttggaccagggctcgagaggcacctccatggtggcatgcagatctttgtgaagacatagaatgctcaagaacatgtgatgattggagggtaacaatcctcaccaagatcctcaccgaataaacccacaagacccccccggcaagatccttgccgaggacggcaagcgccacggcaagacccttgccgggccacccaacgagaccctcgccagaggcaaccacgagcccactgccaggcccgcaccaaccaactccccgccgccgttcgcatgcagctgccaacccaaccagctgagcaggcacctgcgtggcaacatgcagcttctaggcccactcagcacacacctgtgtggcggcatgcagatcttcgtggaggctccaccaccgcaccacctcagctgcttgcctgcctacatggcaccgcatgcatcgctggccagggcgcgtgtcgaagcaaggaggagcggcgacggatgggacgggcgtcgctcccgtccccgataaagtgagggacacctaagccatgcat↑gttcatcatgtgagacggactagtcatcatcggtgaacatctccatgttgatcgtatcttccatacgactcatgttcgaactttcggtcccttgtgttccgagtccatgtctgtacatgctaggctcgtcaagttaacataagtgttttgcatgtgtaaatctgtcttacacccattgtatgtggacgttggaatctatcacacccgatcatcacgtggtgcttcgaaacaatgaactt… (C) Assay for the junction between intact *Ppd-B1* gene copies in the ‘Sonora64’/‘Timstein’/‘C591’ allele. Primer positions for this assay are underlined in the sequence below. The junction is shown by a vertical arrow. The TREP 3457 Danae element is to the left of the arrow, the TREP 3173 Derami element is to the right. A 223 bp band is generated when the junction is present. …tcatggttgatgcacttgccggtggattccatctcttcttgggcggatctgtcccatgagtttgttggcgcctttaccggaggtcaccaagctcatggccaggcgagtgatttacacatcacccctcagaaggaatgagaaaacttgcgcaagtacatccaaaggttcagccgggtgcagtacaacatccccgatgttcatcccgccgtcgtgatcagtgtgttccattagaatgtgcgcaaccgcaagatgcgtgaagagctagcgatgaacaa↑aagttaaattttcatgaagtgaaataaaaggtgtgttaacgtgcccatgccatctattttcaaaaataaataaataatctttaagaaaagttaaatgcataaaaaagtccaatagtttacatatacattggctcaatatagtggtgaggcatatttatttatattactcatggtcttt… Descriptions of repeat sequences and TREP numbers in (A) to (C) follow Figure 3 in the main text.
(TIF)

Figure S3 **QTL Cartographer plot of days to ear emergence in ‘Malacca’×‘Hereward’ DH lines.** Plants were vernalized for 4 weeks (5°C; 8 h light) and grown in lit glasshouse (18 h light). For each DH line the score was the mean of three plants. One region exceeded the significance threshold (black line at LOD 2.5) and this was on a 20 cM linkage group containing marker *barc151* previously shown to be linked to *Vrn-A1* [35]. When *Vrn-A1* was scored qualitatively based on copy number scores from the TaqMan® assay (Figure 6B in the main text) the QTL peak coincided with *Vrn-A1* position.
(TIF)

Figure S4 **Example trace files of directly sequenced PCR amplicons from the region of** ***Vrn-A1*** **exon 4 containing the C/T variants.** ‘Hereward’ and ‘Malacca’ had a C/T double peak (arrowed) while ‘Claire’ and ‘Chinese Spring’ had only the C form.
(TIF)

Figure S5 **C and T variants in exon 4 of** ***Vrn-A1*** **.** (A) Predicted amino acid sequence of part of the K box region of VRN-1 and related MADS-box genes. Protein sequences are from related MADS-box genes [40] plus the exon 4 C and T forms of VRN-A1. The T form changes a conserved leucine to a phenylalanine (arrowed position). (B) Analysis of the spring allele of *Vrn-A1* from ‘IL369’. Intr1/C/F, Intr1/AB/R, Intr1/A/F2 and Intr1/A/R3 are previously published primers for PCR assays [16]. Additional primer positions from this paper are shown as red arrows with the primer sequences underneath. IL369_F1 is specific to the intact copy and an example trace file of the IL369_F1/IL369_R1 product is shown to the right. IL369_F2/IL369_R1 could amplify from the intact and deleted copies but the much larger amplicon from the former was not observed. An example trace file from the IL369_F2/IL369_R1 product is shown to the right. The C/T variant is highlighted.
(TIF)

File S1 **Quantification of** ***Ppd-B1*** **expression.**
(DOC)
