# Supplementary figures and images for: Copy Number Variation Affecting the Photoperiod-B1 and Vernalization-A1 Genes Is Associated with Altered Flowering Time in Wheat (Triticum aestivum)
Source: PLoS One. 2012 Mar 22;7(3):e33234. doi: 10.1371/journal.pone.0033234 (PMC3310869; doi:10.1371/journal.pone.0033234)

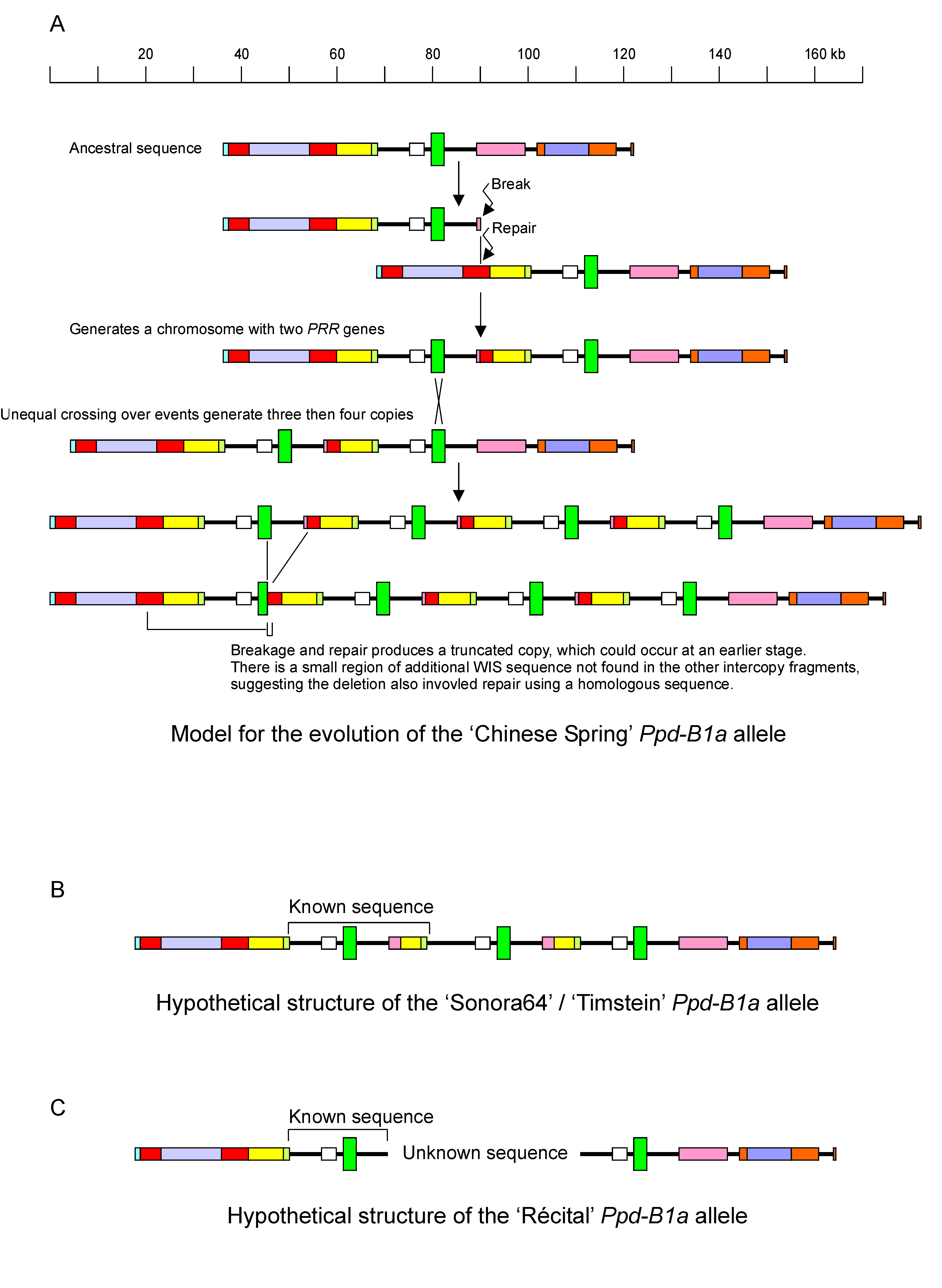

Supplement: Figure S1 — A model for the origin of the ‘Chinese Spring’ Ppd-B1a allele and hypothetical structures of the ‘Sonore64’/‘Timstein’ and ‘Récital’ Ppd-B1a alleles. (A) The ‘Chinese Spring’ Ppd-B1a allele. (B) Hypothetical structure of the ‘Sonora64’/‘Timstein’ Ppd-B1a allele. (C) Hypothetical structure of the ‘Récital’ Ppd-B1a allele. Ppd-B1 copies are shown as large dark green rectangles (exons plus introns), the solid black line shows upstream and downstream regions and small coloured rectangles show transposable elements as in Figure 3 of the main text. (TIF) [file pone.0033234.s001.tif]

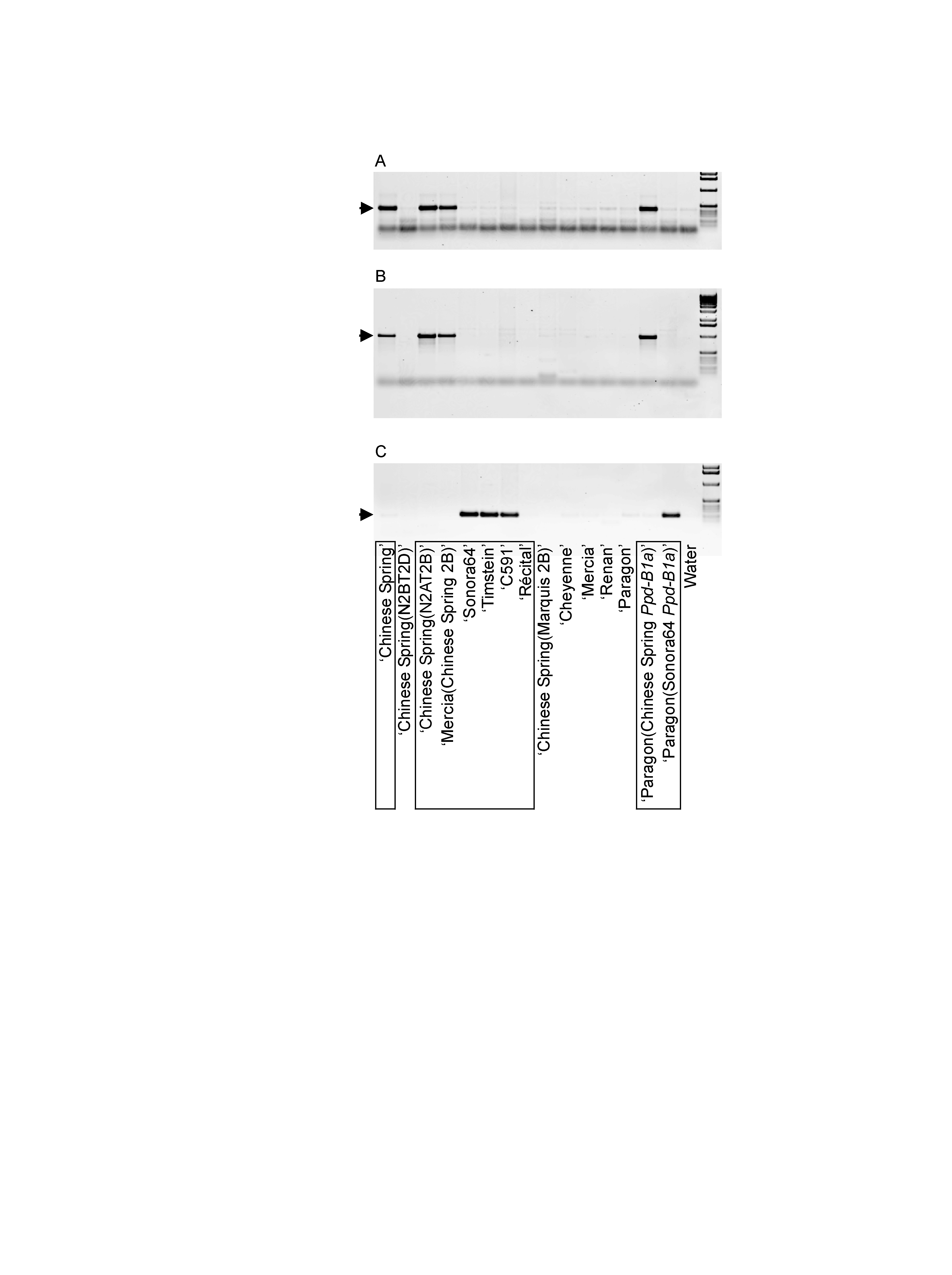

Supplement: Figure S2 — PCR assays detecting junction sequences in Ppd-B1a (day neutral) alleles and sequences of the junction regions. (A) to (C) Reverse colour images of PCR products from genotypes with known Ppd-B1 alleles. Genotypes with a known Ppd-B1a allele are boxed. ‘Chinese Spring(N2BT2D)’ is nullisomic for chromosome 2B (no Ppd-B1 gene is present) and tetrasomic for chromosome 2D. ‘Chinese Spring(N2AT2B)’ is nullisomic for chromosome 2A and tetrasomic for chromosome 2B. (A) Assay for the gene/transposon junction (vertical arrow) in the truncated Ppd-B1 copy of ‘Chinese Spring’. Primer positions for this assay are underlined in the sequence below. Ppd-B1 gene sequence is to the left of the arrow (exons in uppercase, intron in lowercase) and the TREP 3161 WIS element is to the right of the arrow. A 425 bp band is produced when the junction is present. … TAACTGCTCCTCACAAGTGCCGGAAGGGAAAGACGCCGACCGTGAGAACGCCATGCCATATCTTGAGCTGAGCCTAAAGAGGTCGAGATCGACCACGGAGGGTGCGGATGCGATCCAGGAGGAACAGAGGAACGTCGTGAGACGATCAGACCTCTCGGCATTCACGAGgtgcaaagcataatatcagtgtcctttgtgaatccttaaatcatccatatgttgcatactaaccgttttcattctttgcaagGTACAATACGTGCTCGTTCTCCAATCAAGGCGGGGCAGGGTTCGTCGGGAGCTGTTCGCCCA↑cgtgactgccaagcgttcataacgtcttggttctatgggatgggtgcttcacctagcggtccttctaggacatatgctttcttggcagctatgaggatgatcctcaggttccgg . The same primers were used for RT-PCR where a 343 bp product was produced from cDNA. The product was cloned and sequenced, confirming that intron 6 was correctly spliced. The transcript has a stop codon (double underline) close to the break point which gives a predicted protein lacking a CCT domain. (B) Assay for the junction between intact Ppd-B1 gene copies in the ‘Chinese Spring’ allele. Primer positions for this assay are underlined in the sequence below. The left primer spans the junction between Ppd-B1 gene sequence and the TREP 3457 Danae element. The junction (vertical arrow) marks the start of the TREP 3161 WIS element sequence which contains the right primer. A 994 bp band is generated when the junct [file pone.0033234.s002.tif]

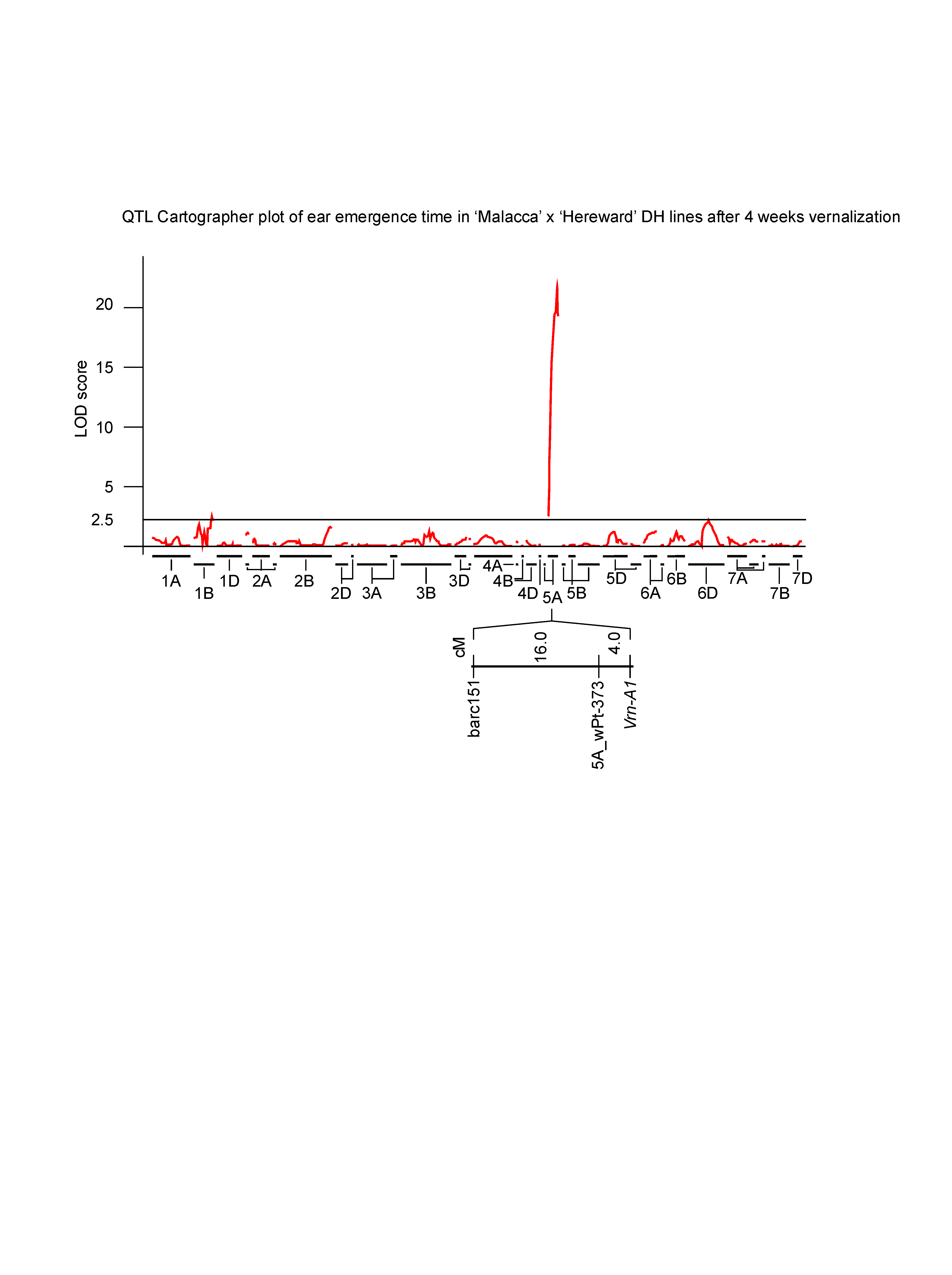

Supplement: Figure S3 — QTL Cartographer plot of days to ear emergence in ‘Malacca’×‘Hereward’ DH lines. Plants were vernalized for 4 weeks (5°C; 8 h light) and grown in lit glasshouse (18 h light). For each DH line the score was the mean of three plants. One region exceeded the significance threshold (black line at LOD 2.5) and this was on a 20 cM linkage group containing marker barc151 previously shown to be linked to Vrn-A1 [35]. When Vrn-A1 was scored qualitatively based on copy number scores from the TaqMan® assay (Figure 6B in the main text) the QTL peak coincided with Vrn-A1 position. (TIF) [file pone.0033234.s003.tif]

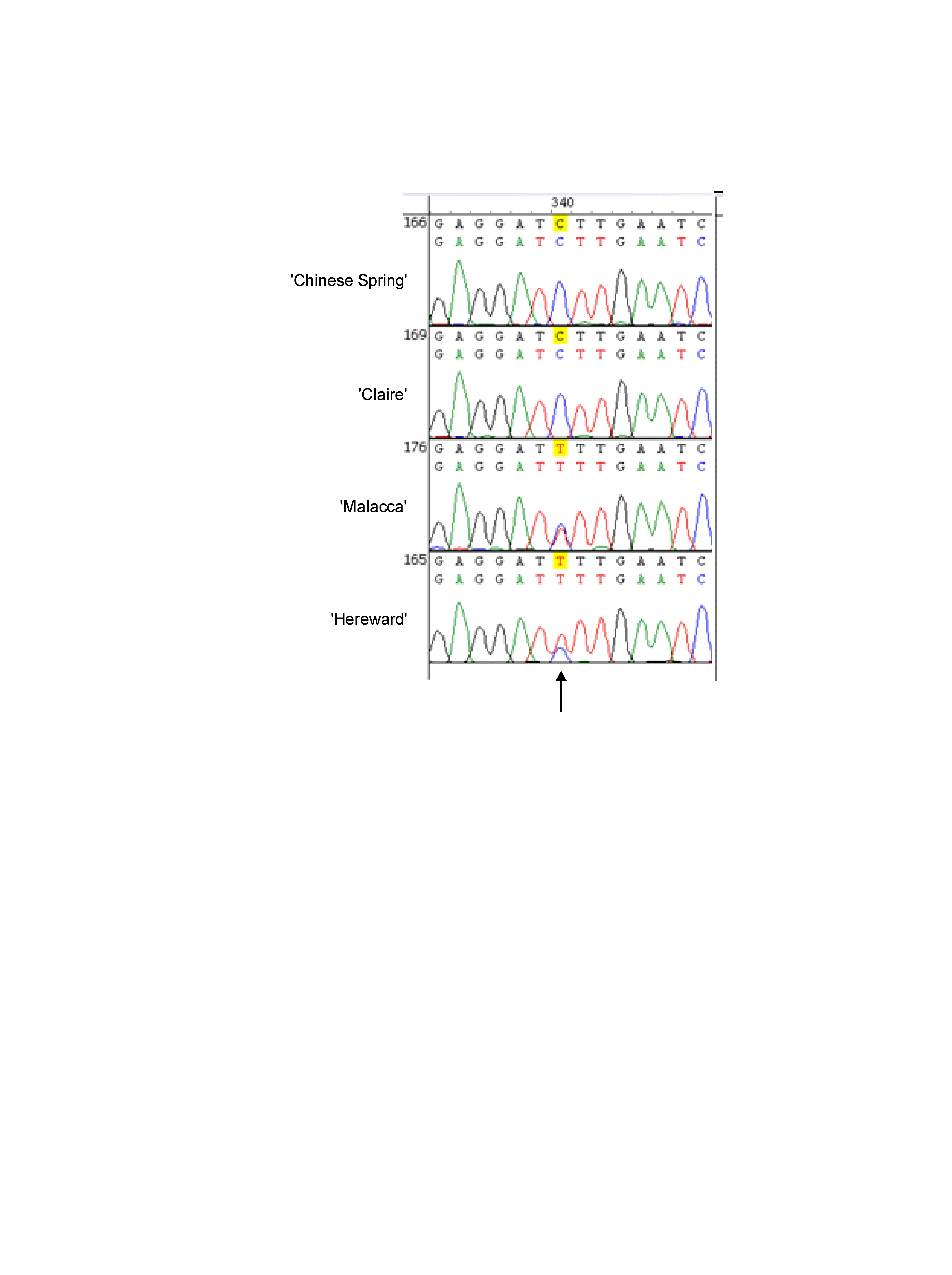

Supplement: Figure S4 — Example trace files of directly sequenced PCR amplicons from the region of Vrn-A1 exon 4 containing the C/T variants. ‘Hereward’ and ‘Malacca’ had a C/T double peak (arrowed) while ‘Claire’ and ‘Chinese Spring’ had only the C form. (TIF) [file pone.0033234.s004.tif]

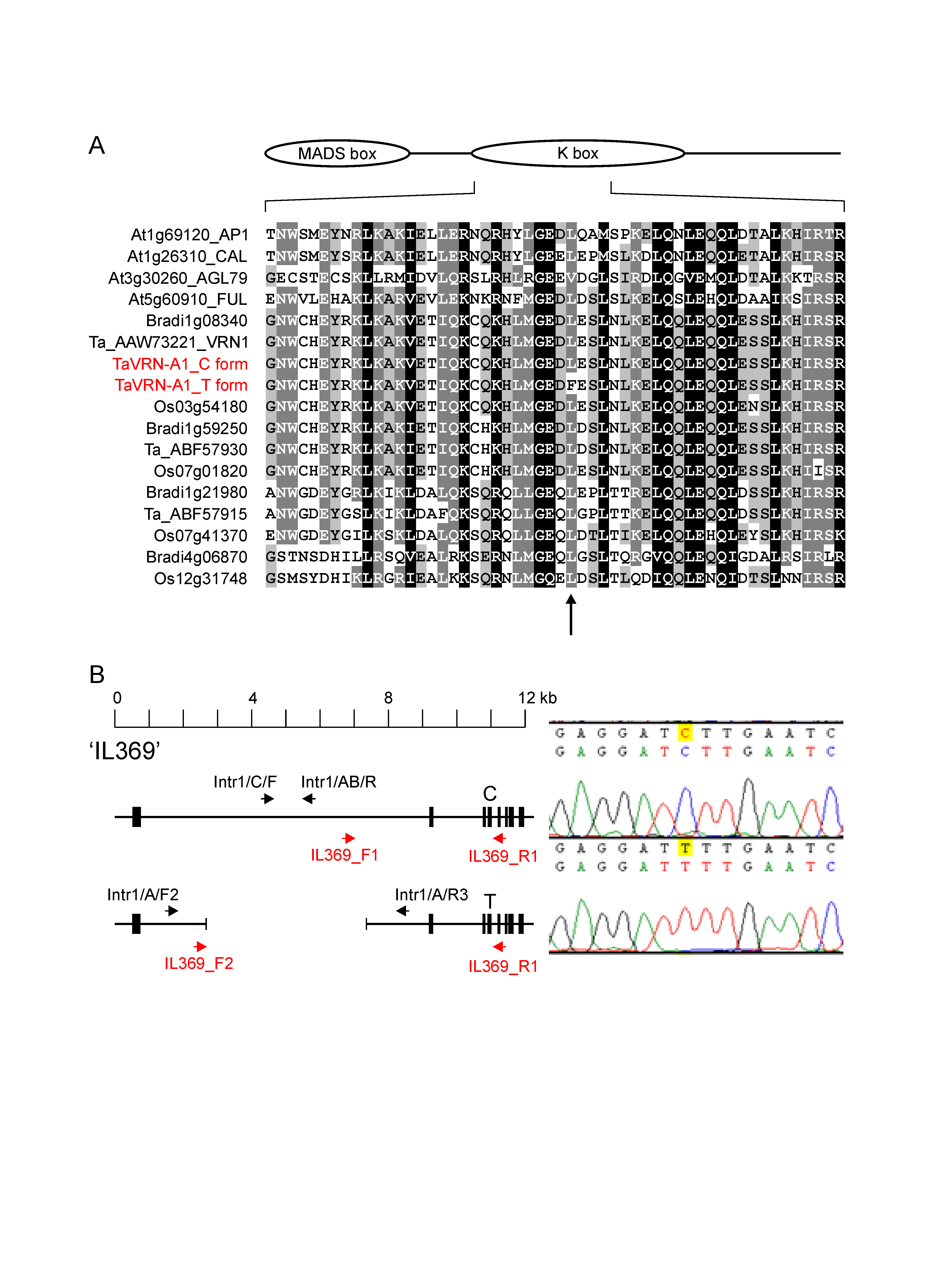

Supplement: Figure S5 — C and T variants in exon 4 of Vrn-A1 . (A) Predicted amino acid sequence of part of the K box region of VRN-1 and related MADS-box genes. Protein sequences are from related MADS-box genes [40] plus the exon 4 C and T forms of VRN-A1. The T form changes a conserved leucine to a phenylalanine (arrowed position). (B) Analysis of the spring allele of Vrn-A1 from ‘IL369’. Intr1/C/F, Intr1/AB/R, Intr1/A/F2 and Intr1/A/R3 are previously published primers for PCR assays [16]. Additional primer positions from this paper are shown as red arrows with the primer sequences underneath. IL369_F1 is specific to the intact copy and an example trace file of the IL369_F1/IL369_R1 product is shown to the right. IL369_F2/IL369_R1 could amplify from the intact and deleted copies but the much larger amplicon from the former was not observed. An example trace file from the IL369_F2/IL369_R1 product is shown to the right. The C/T variant is highlighted. (TIF) [file pone.0033234.s005.tif]
