# Supplementary material for: Copy Number Variation Affecting the Photoperiod-B1 and Vernalization-A1 Genes Is Associated with Altered Flowering Time in Wheat (Triticum aestivum)
Source: PLoS One. 2012 Mar 22;7(3):e33234. doi: 10.1371/journal.pone.0033234 (PMC3310869; doi:10.1371/journal.pone.0033234)
Supplement: File S1 — Quantification of Ppd-B1 expression. (DOC) [file pone.0033234.s006.doc]

**SUPPORTING INFORMATION**

**Quantification of *Ppd-B1* expression**

Total *Ppd-B1* cDNA was quantified using an Opticon 2 real-time PCR instrument (Genetic Research Instrumentation; [http://www.gri.co.uk](http://www.gri.co.uk/)) with SYBR Green I (Molecular Probes Inc., USA) as a fluorogen. Reactions included 10μl of 2× Promega GoTaq qPCR Master Mix (Promega Corp; [http://www.promega.com](http://www.sigmaaldrich.com/)), 5pmol of each primer, and an aliquot of each cDNA in a total volume of 20μl. Reaction conditions were [95°C 10min; (95°C 15sec, 54°C 15sec, 72°C 40sec] for 40 cycles, followed by a melting curve with 0.2°C steps between 60 and 95°C, and a final polymerization at 72°C for 10min. Optical read temperatures were set such that primer dimers and non-specific products were melted (as determined by melting curve analysis) when reads were taken.

Fluorescence data were collected and analyzed using Opticon Monitor v2 software (BioRad). The fluorescence threshold was set as close to 0.1 absolute units as possible whilst ensuring it was within the exponential phase of all reactions. Ct values were analyzed in Microsoft Excel®. ΔCt values were generated by subtracting the minimum Ct within each assay plate from each sample in turn. Relative expression levels were calculated by expressing as the efficiency value of the PCR reaction *E^-^*^ΔCt^. *E* values were determined from the average rate of fluorescence increase during the exponential phase across all samples in the plate. Target gene expression was normalized against 18S rRNA levels within samples.
